# Challenges to the Long‐Term Safety Claims of Extremely Low‐Dose Amiodarone: Pulmonary, Thyroid, Hepatic, and Statistical Concerns

**DOI:** 10.1002/joa3.70174

**Published:** 2025-08-20

**Authors:** Malik Abdul Rehman Rasheed, Maham Ejaz, Muhammad Furqan Ahsan, Elham Shenawa

**Affiliations:** ^1^ Baqai Medical University Karachi Pakistan; ^2^ Services Institute of Medical Sciences Lahore Pakistan; ^3^ Balkh University Faculty of Medicine Balkh Afghanistan

**Keywords:** amiodarone, hepatic safety, pulmonary toxicity

Dear Editor,

The recent article by Yoshida et al. on the long‐term safety of extremely low‐dose amiodarone (50 mg daily) in patients with persistent atrial fibrillation provides valuable insights into potential rhythm control strategies for a challenging patient population [1]. However, while the study highlights the apparent safety in a select cohort, its conclusions regarding the overall safety and efficacy may be overstated, overlooking key risks supported by contemporary evidence. Herein, we present substantial objections that challenge the findings on scientific, clinical, and statistical grounds.

The assertion of pulmonary safety with extremely low‐dose amiodarone underestimates the risk of interstitial lung disease (ILD), even at low doses. The study reports no clinical pulmonary toxicity; yet this may reflect insufficient power or follow‐up to detect subtle, cumulative effects. Clinically, amiodarone's phospholipidosis and oxidative stress mechanisms can manifest as ILD irrespective of dose, particularly in long‐term use, contradicting the claim of negligible risk. This is evidenced by Tsaban et al. (2024), who demonstrated a trend toward increased ILD risk with low‐dose amiodarone in a nationwide atrial fibrillation cohort, highlighting a relative risk elevation that warrants caution beyond the study's optimistic view [2]. Hence, regarding negligible pulmonary risk with extremely low‐dose amiodarone, emerging evidence underscores the need for heightened caution regarding ILD, advocating for more rigorous, long‐term monitoring in clinical practice to mitigate potential adverse outcomes.

The study inadequately addresses thyroid toxicity, concluding minimal side effects despite amiodarone's well‐known iodine content disrupting thyroid homeostasis. Scientifically, even low doses can lead to cumulative iodine overload, causing hypothyroidism or thyrotoxicosis through destructive or autoimmune pathways, which the study's monitoring may have missed in longer horizons. This challenges the safety profile, as clinical progression can be insidious and impact quality of life. Contradicting evidence comes from Guðjónsson et al., reporting a 40% incidence of thyroid dysfunction after 5 years of amiodarone exposure in a nationwide study, far exceeding prior estimates and emphasizing dose‐independent risks in prolonged therapy [3].

Hepatic safety is overstated, as the study dismisses liver risks without comprehensive enzyme or imaging surveillance beyond baseline. Amiodarone's lipophilic nature promotes hepatic accumulation, fostering steatosis or fibrosis via mitochondrial toxicity, even at low doses, which could explain unreported subclinical changes. Clinically, this poses risks in comorbid patients, undermining the conclusion of broad tolerability. This is countered by Lv and Zhao, who documented increased hepatic iodine density and potential damage via dual‐energy CT in patients on low‐dose oral amiodarone for atrial fibrillation, indicating early toxic effects not captured in the current study [4].

The statistical limitations, including the small sample size (*n* = 100), hinder reliable inference on rare adverse events, inflating type II error and limiting generalizability beyond Japanese patients with lower body mass. The study's quartile analysis and ROC curve for KL‐6 prediction lack robust multivariable adjustment for confounders like age or comorbidities, potentially biasing safety conclusions. This statistical fragility is highlighted by Kwok et al., who noted that small case numbers in amiodarone toxicity studies restrict predictive modeling and risk factor analysis, urging larger cohorts for accurate safety assessments [5].

In summary, these objections—spanning pulmonary, thyroid, and hepatic risks, as well as statistical shortcomings—reveal inconsistencies with established evidence, suggesting that the study's safety claims may not hold universally. I urge the authors to address these discrepancies, perhaps through expanded follow‐up data. Future research should prioritize larger, more diverse multicenter trials with advanced biomarkers to better elucidate the risk–benefit profile of low‐dose amiodarone.

## Author Contributions

**Malik Abdul Rehman Rasheed:** conceptualization, investigation, writing – original draft. **Maham Ejaz:** investigation, writing – review and editing. **Muhammad Furqan Ahsan:** investigation, writing – review and editing. **Elham Shenawa:** conceptualization, supervision, writing – review and editing. All authors read and approved the final manuscript.

## Ethics Statement

The authors have nothing to report.

## Conflicts of Interest

The authors declare no conflicts of interest.

## Data Availability

Data sharing not applicable—no new data generated; or the article describes entirely theoretical research.
